# Variability of grass pollen allergy symptoms throughout the season: Comparing symptom data profiles from the Patient's Hayfever Diary from 2014 to 2016 in Vienna (Austria)^☆^

**DOI:** 10.1016/j.waojou.2021.100518

**Published:** 2021-02-27

**Authors:** Maximilian Bastl, Katharina Bastl, Lukas Dirr, Markus Berger, Uwe Berger

**Affiliations:** Department of Oto-Rhino-Laryngology, Medical University of Vienna, Austria

**Keywords:** Grass pollen allergy, Crowd-sourced symptom data, Electronic symptom diary, Ehealth/mhealth

## Abstract

**Background:**

Grass pollen allergy is the most widespread pollen allergy in the world. It still remains unknown in which aspects and in which extent symptoms from grass pollen allergy differ throughout the grass pollen season, although individual sensitization profiles of persons concerned are known for a long time.

**Methods:**

The crowd-sourced symptom data of users of the Patient's Hayfever Diary were filtered for significant positive correlated users to grass pollen from Vienna (Austria) during the respective grass pollen seasons from 2014, 2015, and 2016. These symptom data were the foundation for 3 statistical approaches in order to examine different sections of the grass pollen season defined either by grass pollen data, phenology (grass species determination in the field), or symptom data itself.

**Results:**

Results from all 3 approaches are similar and come to the same major conclusion. The symptom peak of most users is observed in the second section of the grass pollen season (70%), followed by the first section (20%), and with the least user numbers (10%) the third section. The profiles from single users entering data for all 3 years under study are robust and show a comparable behavior from year to year.

**Conclusion:**

Grass taxa such as *Arrhenatherum*, *Festuca*, and *Lolium* seem to induce the highest symptom severity in most users during the second section of the grass pollen season. *Poa* and *Dactylis* are the main triggers for the first section of the grass pollen season. The flower of *Phleum* und *Cynodon* is documented for the last section of the grass pollen season. Crowd-sourced symptom data is the prerequisite for personal pollen information to consider the individuality of grass pollen allergy sufferers. Phenological monitoring is needed to provide information on specific grass taxa of importance to allergic persons.

## Introduction

Pollen allergy is a global health problem^1^ and affects a significant percentage within the population of industrialized countries ranging from 5 to 30%.^2^ The frequency of pollen allergies is still presumed to rise^3^ as well as its impact on the health care system and other socioeconomic factors.^1^^,^^4^ In Austria about 1 million people out of 8 million inhabitants suffer from pollen allergies.^5^ The most common pollen allergy in Austria including the highest sensitization rates (more than 50%) can be attributed to grass pollen.^6^ Grass pollen allergy itself is one of the most common pollen allergies worldwide with sensitization rates up to 30% depending on climate and region.^7^^,^^8^ The high sensitization rates to grass pollen are related to the nearly ubiquitous distribution of grasses. The sweet grass family (Poaceae) is one of the largest plant families worldwide^9^ and covers up to 40% of Earth's vegetation.^10^ Moreover, 11 groups of grass pollen allergens have been identified up to now,^7^ and extensive cross-reactivity is documented among allergens of different grasses.^11^ On the other hand, recent studies indicate that individual grasses show differences in their IgE and IgG reactivity as well as in protein content^12^ and variable cross reactivity on T-cell levels suggesting a multiple allergen system contributing to the burden of allergic disease.^13^ Other studies focusing on the phenological and aerobiological distribution of different grasses at specific localities support the hypothesis of a multiple allergen system since a variety of grasses contribute to the grass pollen season and the symptomatic burden of grass pollen allergy sufferers.^14^, ^15^, ^16^, ^17^ Triggering allergic symptoms is a complex process and individual pollen allergy sufferers might react in different ways, not only during the pollen season.^18^ The most effective way to evaluate different profiles during the grass pollen season is symptom data. The use of symptom data is performed regularly in clinical trials and confirmatory studies to compare symptoms and pollen concentrations with a combination of symptom and medication scores or rhinitis quality of life scores when immunotherapy is administered.^19^ However, patient recruitment and administration is a laborious and cost consuming task and the data may not be used in scientific studies in some cases due to reduced adherence rates or a small cohort size. Therefore, the value of electronically generated symptom data of freely available symptom diaries increased in recent years, and online diaries became more popular also for scientific questions since the amount of data sets and cohort sizes are larger and the adherence rates comparable to the those of clinical studies.^20^, ^21^, ^22^ In this study, the individual profiles of more than 200 crowd-sourced users identified as grass pollen allergy sufferers entered data into the Patient's Hayfever Diary (PHD) during the years 2014–2016. This data was evaluated to examine possible seasonal differences in the symptom data profiles during the grass pollen season in relation to pollen concentration peaks throughout the season, the pollination period of different grass species, or the symptom load in general.

## Methods

### Phenology

**Fig. 1:**
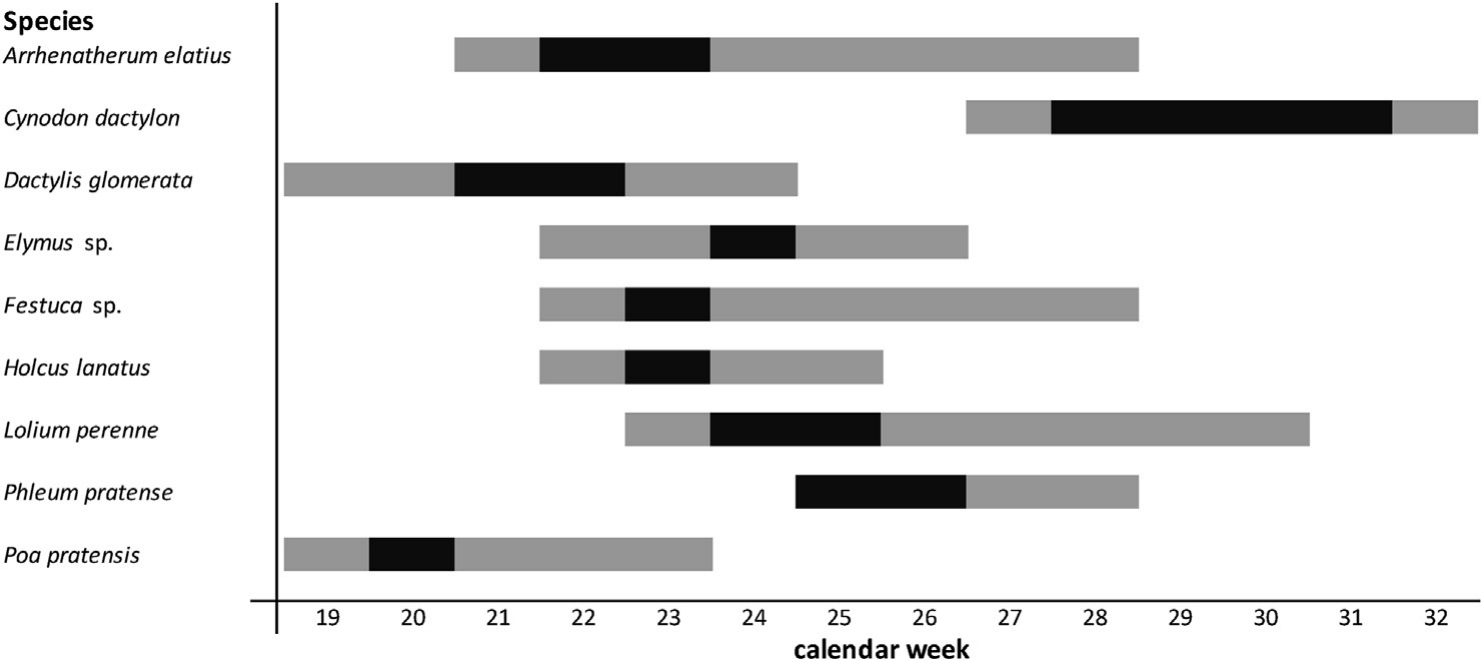
Pollination periods of the most abundant grass species in Vienna in the respective calendar weeks averaged for the years 2014–2016. The black bar represents the full flowering stage, whereas the grey bars describe the start and the end of the flowering period.

Phenological observations and grass species identification were undertaken once to twice a week in 3 different locations in Vienna during the years 2014–2016. Extensive observation sites in different urban habitats were selected to cover a representative range of grass species. Three large observation sites with a total area larger than 80 000 m^2^ were observed to assess phenological data. The western site “Steinhofgründe” represents a natural habitat since it is defined as a natural monument and is in close distance to the surrounding woods of Vienna. The second location “Neue Donau/Wasserpark” is more urbanized and consists of a public park, riverside, as well as rail track vegetation. The third observation area is located in the garden of the Central Institution for Meteorology and Geodynamics (ZAMG), in a suburban city area and next to the local pollen monitoring station of Vienna. All selected observation areas are located within the city borders of Vienna and are in close distance to the pollen monitoring station. Moreover, they are representative regarding grass species distribution^23^ and performed well in recently conducted studies.^15^^,^^16^ The phenological observations were studied by the use of the random field approach, which includes several random fields with surfaces of approximately 4 m^2^ per location. However, these random fields could be set wider apart due to governmental mowing activities. Five different phenological phases have been defined to determine the pollination periods of each grass species.^15^^,^^16^ These phases were translated into international Biologische Bundesanstalt, Bundessortenamt und Chemische Industrie scale (BBCH-scale) phenological phases.^24^ Only more than 25 individuals per grass species and defined area were examined to evade observing poorly distributed grasses at the respective surface.^14^ The phenological phases of the most distributed grass species from years 2014–2016 were averaged to define the respective time period for the user selection of the PHD (Fig. 1). Detailed information regarding the phenological phases and the most important grass species in Vienna can be found in Kmenta et al.^15^^,^^16^

### Pollen measurements

Daily pollen data were assessed with a volumetric pollen and spore traps of the Hirst design^25^ during the whole observation period from 2014 to 2016 in Vienna. The collected data was evaluated according to the minimum recommendations of the European Aeroallergen Society^26^ to ensure high data quality. The main pollination period of the grass pollen seasons 2014–2016 was defined by applying the standardized season definition of the European Aeroallergen Network (EAN). Hence, the start of the season is defined as the day when 1% of the cumulative annual total grass pollen amount is reached and the season ends with 95% of the total annual pollen count. The start dates, end dates, as well as the duration of the pollen seasons and the annual pollen integrals (APIn,^27^) are summarized in Table 1 for an overview of the grass pollen seasons. The course of the grass pollen seasons from 2014 to 2016 is included in Supplementary File 1.

**Table 1:** Start dates, end dates, duration and Annual Pollen Integral of the grass pollen seasons 2014–2016 in Vienna as well as the mean values are summarized. The respective start and end of the season is displayed in addition to the dates the day of the year (doy)

Year	Start date (doy)	End date (doy)	Duration (in days)	Annual Pollen Integral (APIn)
2014	30.04.2014 (120)	19.07.2014 (200)	81	2154
2015	30.04.2015 (120)	22.07.2015 (203)	84	3467
2016	01.05.2016 (121)	06.08.2016 (218)	98	2677
Mean	30.04.yyyy (120)	26.07.yyyy (207)	88	2766

### Crowd sourced data for the individual symptom profiles

The symptom data for the individual grass pollen profiles originates from the PHD (https://www.pollendiary.com). This tool is a free web-based online diary which records the symptoms of users suffering from pollen allergies and was already used in several scientific studies eg, Refs. 18,28, 29, 30. Users fill in a validated questionnaire and indicate the symptom severity of eyes, nose, and lungs including medication use. A total symptom and medication score can be calculated with this basic information. In addition, the questionnaire asks for a zip code to dedicate the users to a biogeographical region and assign the symptom data to a respective pollen monitoring station. Symptom score calculation and the user pool in Austria is described in detail in Bastl et al.^20^ Moreover, the PHD fulfills the latest European Union (EU) regulation on data privacy (regulation EU 2016/679) and adheres to the General Data Protection Regulation, Directive 95/46/EC.^20^ In this study the symptom data of the PHD was filtered to specify user profiles as “grass pollen allergy sufferers” and were used as a “proxy” to understand symptoms. Hence, only users reporting symptoms in Vienna with a significant positive background correlation to grass pollen (positive correlation factor, significance rates <0.05) in the grass pollen seasons of 2014, 2015 and 2016 and at least 15 data entries (minimum of 15 days) during the main grass pollen season were included into the evaluation.

### Statistical analysis

The PHD includes an automated background correlation service. Therefore, the correlation significance rates for all users included in this study attain values between 0.01 and 0.05 since they were already filtered according to a significant positive background correlation to grass pollen. All statistical computations were performed in the software environment R 3.6.1.^31^ In addition, the R package data. table^32^ was used for data manipulation tasks and the R package ggplot2^33^ for visualizations. The kNN function from the Rpackage VIM^34^ was employed to apply the nearest neighbor imputation. Three different methods have been performed to separate the grass pollen season into different phases (sections) and to classify the users within the grass pollen season.

In the first approach the season was split into 3 sections according to the annual pollen integral (APIn). The first section ranges from zero to one third of the APIn, the second one from one-third to two-thirds of the APIn, and the third from two-thirds to the APIn.

The second approach split the season according to dates derived from phenological observations. The first section started with the beginning of the early flowering grasses (defined by *Poa pratensis* and *Dactylis glomerata*), the second section included the dates of the grasses flowering throughout the main grass pollen season (defined by *Arrhenatherum elatius*, *Festuca* sp. and *Lolium perenne*), and the third section included late flowering grasses (defined by *Phleum pretense*, *Cynodon dactylon*).

Moreover, the total SLI sum of each user was divided into 3 sections separately. Hence, the first phase of the season for 1 person is until one-third of the total SLI sum is reached, the second phase is until two-thirds are reached, and the third phase is the remaining SLI of the season. Missing values of users during the season are problematic for this approach and an impute is needed (missing values have to be filled beforehand). A widely used imputation is k nearest neighbor imputation. K similar observations (based on a distance function) are aggregated to get an estimated value for the missing SLI data points. Hence, the day of the season and the pollen concentration as distance variables were used to compute the median of the 3 nearest data points to fill such gaps (k was set to 3). With an analysis of variance (ANOVA) the significant influence of the respective sections on the SLI was confirmed in addition for each approach (see Supplementary File 2).

## Results

### Participants

In total, the profiles of 267 PHD users from Vienna have been included in the evaluation from the years 2014–2016. The user numbers, as well as age groups, ethnicity and sex were different depending on the year and the course of the grass pollen season. 23 PHD users entered data in all 3 years of the study period. In total 12 users entered data throughout the whole grass pollen season (6 in 2014, 3 in 2015, and 3 in 2016), whereas only 5 users entered the minimum data amount of 15 entries during the season (0 in 2014, 3 in 2015, and 2 in 2016). The average number of days entered by the PHD users amount up to 49 days during the grass pollen season.

### Descriptive data

The year 2014 was characterized by an average grass pollen season with a duration of 81 days (see Supplementary File 1; Table 1) and recorded 104 PHD users with a significant positive correlation to grass pollen. In the year 2015 the user numbers slightly decreased to 89 PHD users although the grass pollen season was more intense with pollen concentrations above the 5-year average and a duration of 84 days (see Supplementary File 1; Table 1). The year 2016 recorded the longest seasonal duration of 98 days in combination with an about average performing pollen season (see Supplementary File 1) and the lowest PHD user numbers (74 users).

### Main results

Three statistical approaches were performed to apply the PHD users to specific time periods (sections) according to their highest symptom severities during the grass pollen season. The grass pollen season was divided into 3 sections based on the annual pollen integral (APIn), the symptom load index (SLI), and the phenological observations within the pollen season (Table 2). The majority of the users (60.3% in the APIn calculation, 54.7% in the SLI calculation, and 70.8% in the phenology calculation) experienced the highest symptom load in the second section of the grass pollen season which can be assigned to the main pollination period. More than 20% of the users of the PHD experience the highest symptoms in the beginning of the grass pollen season before the main pollination period in all calculations (Table 2). The smallest user fraction recorded the highest symptom severity in the period after the main pollination period ranging from 21% (SLI) to 8.6% (phenology) (Table 2). The allocation of the PHD users into the different phenological phases includes the most important grass species in Vienna and was investigated on a yearly basis (Table 3). The yearly investigation shows a comparable picture which confirms that more than 20% of the users experience the highest symptoms in the beginning of the season, except for the year 2015 when this amount was only 16.9% (Table 3). The highest number of users entered the highest symptoms in every year during the main pollination period and user numbers ranged from 64.4% in 2014 to 78.6% in 2015 (Table 3). The lowest user numbers recorded high symptom data in the period after the main pollination period and ranged from 4.5% in the year 2015 to 12.5% in the year 2014. The profiles of the PHD users which entered data in all years were considered comparable in all approaches.

**Table 2:** Division of the PHD users into different parts during the grass pollen season based on the annual total pollen amount (APIn), the phenology and the SLI in Vienna during the years 2014 and 2016

Seasonal parts	Total users per part (APIn)	% of users (2014–2016)	Total users per part (phenology)	% of users (2014–2016)	Total users per part (SLI)	% of users (2014–2016)
1	58	21.7%	55	20.6%	65	24.3%
2	161	60.3%	189	70.8%	146	54.7%
3	48	18%	23	8.6%	56	21%

**Table 3:** Division of the PHD users into different parts during the grass pollen season based on phenology in Vienna, visualisation of the different years

Seasonal sections	Year 2014	% of users (2014)	Year 2015	% of users (2015)	Year 2016	% of users (2016)
1	24	23.1%	15	16.9%	16	21.6%
2	67	64.4%	70	78.6%	52	70.3%
3	13	12.5%	4	4.5%	6	8.1%

### Other analyses

In addition to the main results, a subgroup analysis of the 23 PHD users who consistently entered data throughout all pollen seasons was performed to verify the stability of the symptom data profiles in different seasons.

Eight users (35%) presented a robust user profile and were assigned to the same section in all 3 years based on the APIn calculation, whereas the phenological division assigned 9 users (39%) to the same section. Eleven users (48%) showed the same profile in 2 out of 3 years and were assigned to a different section in one of the years using the APIn calculation whereas 12 users (52%) performed the same way in the phenological calculation. It is noteworthy, that the change of a section for different years always concerns successive sections; this means no single user was found reacting in the first and the third section in all 3 years. Hence, 4 users (17%) reveal variable profiles in all years using the APIn division calculation, whereas only 2 users (9%) show variable profiles in all years using the division calculation originating from the phenological observations.

## Discussion

**Fig. 2:**
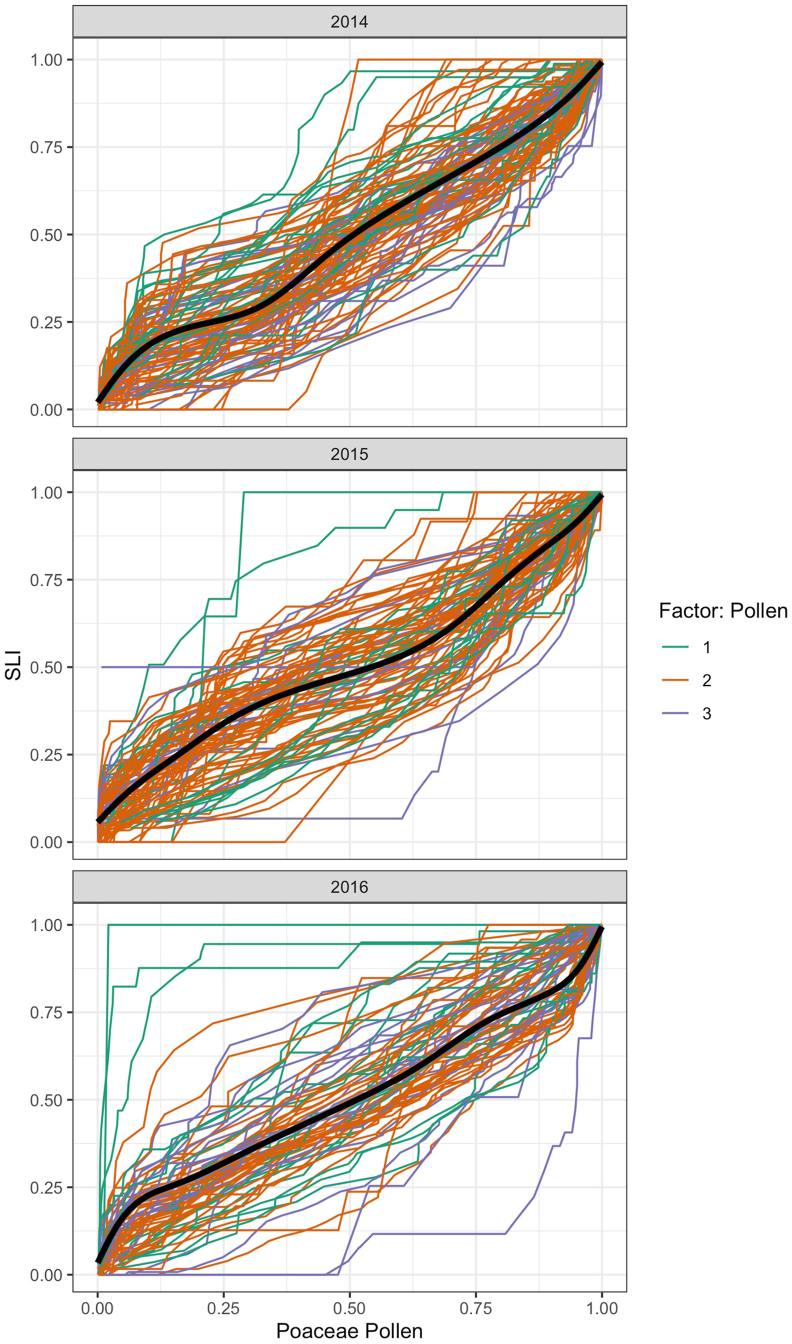
Cumulative sum of the SLI (y-axis) compared to the cumulative sum of the annual pollen integral (APIn) (x-axis). The profiles were divided into three sections based on the annual pollen integral (APIn): 1 = highest symptom data in the beginning of the grass pollen season (green lines); 2 = highest symptom data in the main grass pollen season (orange lines) and 3 = highest symptom data in the end of the grass pollen season (blue lines). The black line represents the average course of all grass pollen allergy sufferers' profiles. Grass pollen allergy profiles above this average can be attributed to pollen allergy sufferers with a fast reaction pattern. Profiles below the average have a slow reaction pattern. (For interpretation of the references to colour in this figure legend, the reader is referred to the Web version of this article.)

**Fig. 3:**
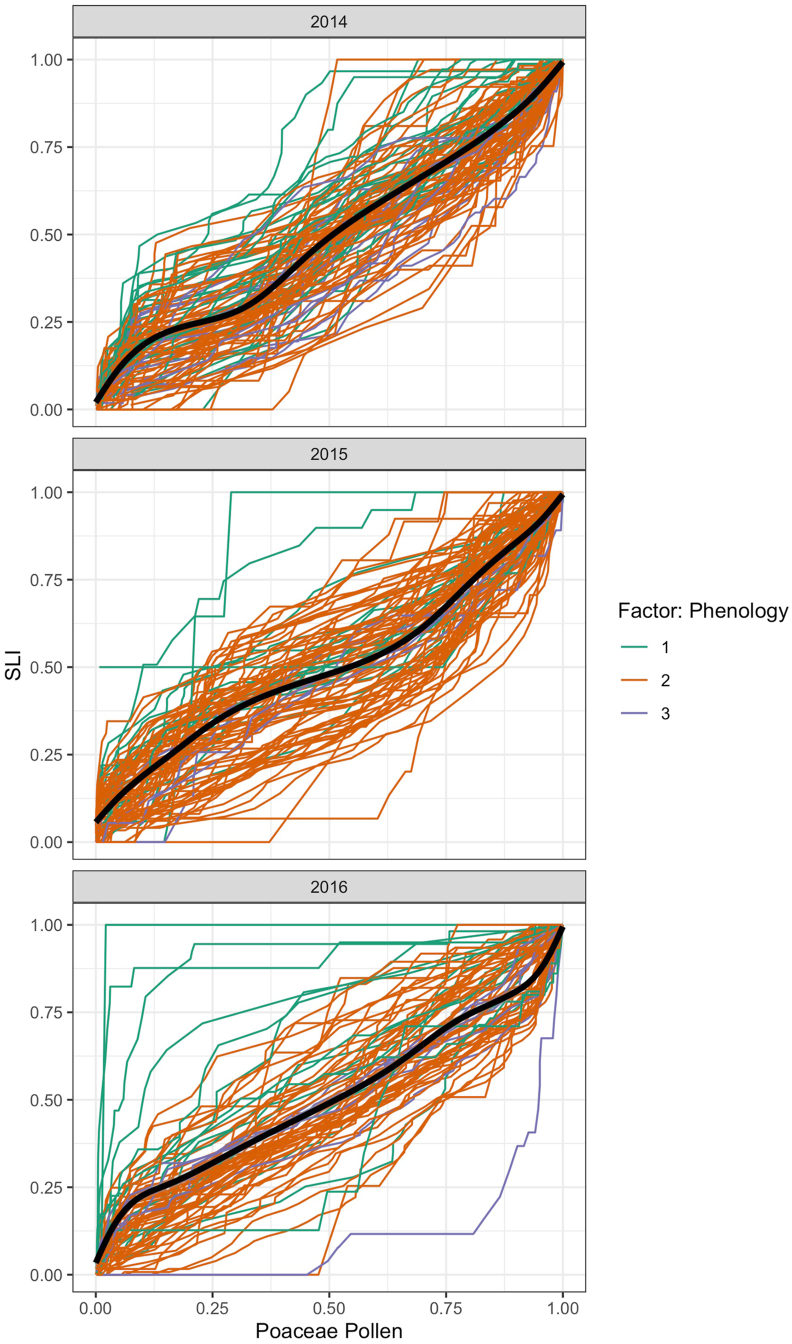
Cumulative sum of the SLI (y-axis) compared to the cumulative sum of the annual pollen integral (APIn) (x-axis). The profiles were divided into three sections based on the phenological observations in Vienna: 1 = highest symptom data in the beginning of the grass pollen season (green lines); 2 = highest symptom data in the main grass pollen season (orange lines) and 3 = highest symptom data in the end of the grass pollen season (blue lines). The lack line represents the average course of all grass pollen allergy sufferers' profiles. Grass pollen allergy profiles above this average can be attributed to pollen allergy sufferers with a fast reaction pattern. Profiles below the average have a slow reaction pattern. (For interpretation of the references to colour in this figure legend, the reader is referred to the Web version of this article.)

**Fig. 4:**
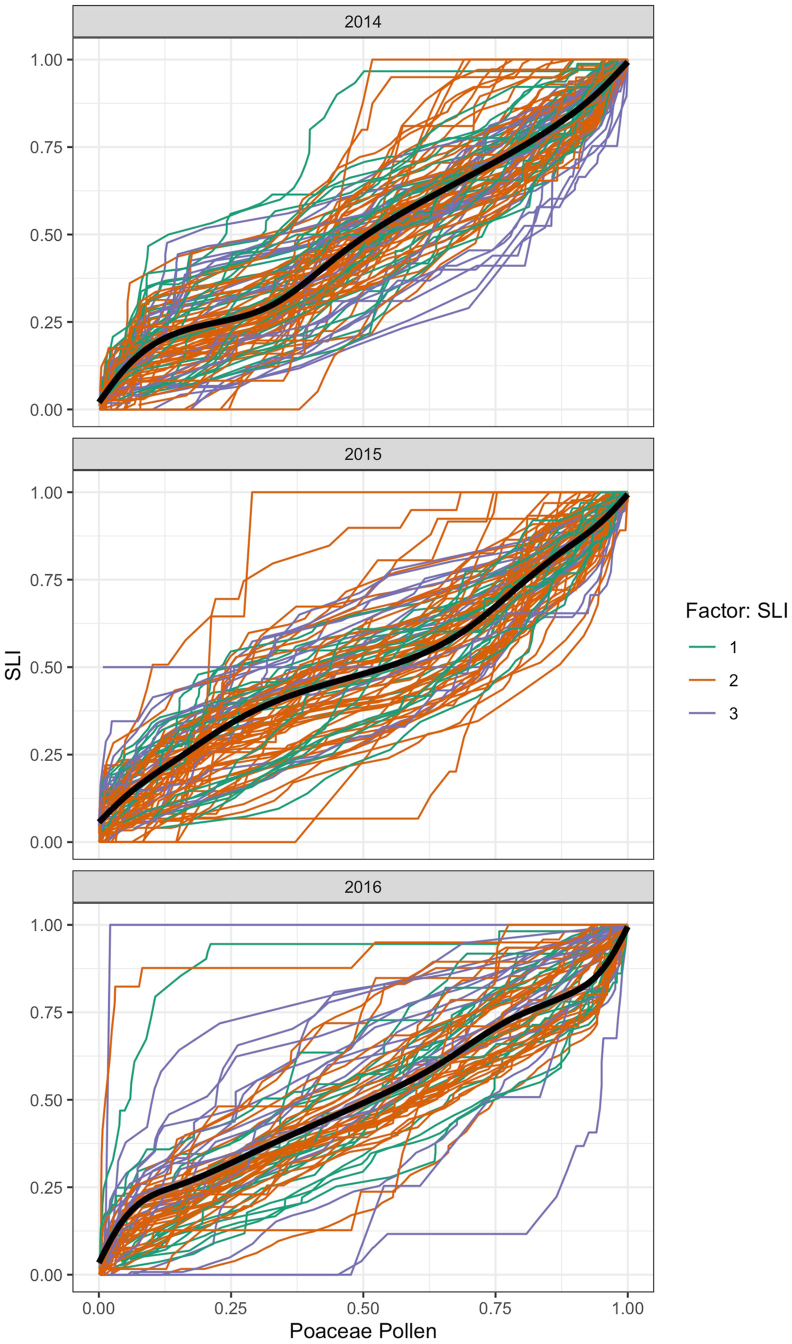
Cumulative sum of the SLI (y-axis) compared to the cumulative sum of the annual pollen integral (APIn) (x-axis). The profiles were divided into three sections based on the Symptom load index (SLI): 1 = highest symptom data in the beginning of the grass pollen season (green lines); 2 = highest symptom data in the main grass pollen season (orange lines) and 3 = highest symptom data in the end of the grass pollen season (blue lines). The black line represents the average course of all grass pollen allergy sufferers' profiles. Grass pollen allergy profiles above this average can be attributed to pollen allergy sufferers with a fast reaction pattern. Profiles below the average have a slow reaction pattern. (For interpretation of the references to colour in this figure legend, the reader is referred to the Web version of this article.)

The results of the study show that grass pollen allergy sufferers have highly individual symptom severity profiles (Fig. 2, Fig. 3, Fig. 4) but the grass pollen season can be divided into 3 sections when taking all analyzed variables into account (APIn, SLI, and phenology). This is of special interest since it is possible to separate different grass species with phenological methods but impossible to assign airborne allergens in the atmosphere since grass pollen are morphologically and their allergens immunologically similar. The sections can be attributed to the 3 main peaks during the grass pollen season which are induced by the local variety of different grass species.^15^^,^^16^ Most pollen allergy sufferers (more than 50%) show the highest symptom severity during the main grass pollen season as defined by the APIn, the SLI, and the phenology approach. This result was expected since the highest pollen concentrations and the highest symptom loads usually appear in the main pollination period. In addition, several other parameters have to be taken into account: Most of the grass species with high IgG and IgE reactivity^12^ are flowering in the field during the main grass pollination period in Vienna as evidenced by the phenological observations^15^^,^^16^ and are an explanation for increased symptom loads due to the possibility of increased allergen content affecting most grass pollen allergy sufferers. Moreover, the main pollination period of the grasses is correlating with the highest ozone concentrations^35^ which may also increase the symptom severity during this time period. Grass pollen concentrations and symptom data are following a linear trend until they reach a plateau.^36^ The main grass pollen season seems to be the time frame when most users are reaching this plateau and show no increase in symptom severity afterwards.

Approximately 20% of the PHD users experience the highest symptom loads in the beginning of the grass pollen season depending on the statistical calculation method. Hence, the symptom plateau is reached earlier. There are several explanations for this reaction pattern. It is known in literature that an increase of symptoms can be recorded in the beginning of the season.^37^ An explanation for this phenomenon could be the sensitization to additional aeroallergens (eg, polysensitization) or the priming effect.^29^^,^^38^ Moreover, an individual reaction to early flowering grasses could be possible in the start of the grass pollen season. The meadow grasses (genus *Poa*) usually indicate the start of the grass pollen season in Europe^15^, ^16^, ^17^ and announce the grass pollen season, whereas *Dactylis glomerata* is also one of the early flowering grasses and shows high IgE and IgG reactivity as well as protein content.^12^ An intense flowering of these grass species could have an effect on the symptom profiles of grass pollen allergy sufferers and explain the high number of users experiencing the highest symptoms in the beginning of the grass pollen season.

Approximately 10% of the PHD users record the highest symptoms in the last section of the grass pollen season. There are several explanations for this result as well. The main pollination period of *Phleum pratense* and *Cynodon dactylon* (Fig. 1)^15^^,^^16^ is located in the last section of the grass pollen season and could increase the symptom load in grass pollen allergy sufferers especially sensitized to these species. Moreover, the pollination period of corn (*Zea mays*) coincides with this timeframe and could have an additional effect on grass pollen allergy sufferers in close vicinity to the growing areas. However, this can be considered less important for the majority of users in Vienna since they move around in urban areas most of the time. In addition, some grass species experience a second flowering period – a rebloom – in the end of the grass pollen season.^15^^,^^16^ Lower symptom severities could be recorded if the users successfully avoided the main pollination period during the season (eg, vacation, medical treatment, or allergen avoidance) but might increase in the end of the season if the users are not aware of this rebloom. Another possible explanation for higher symptom scores in the end of the grass pollen season might be polysensitization to weeds (eg, the Amaranthaceae family or *Artemisia*) or to fungal spores.

The results show a reduction in the numbers of users with significant positive correlation following the seasons 2014–2016. Only a small amount of 23 users entered data in all grass pollen seasons. There are several reasons why users only enter data in some years or seasons. The PHD is a supporting tool for immunotherapy and can be used during this time. If the therapy works and the user does not experience symptoms anymore the entries into the pollen diary will stop. Other explanations are allergen avoidance due to vacation or higher individual thresholds, whereby users experience the seasons as less intense or unknown personal reasons.

Another interesting outcome is that user profiles are more or less stable in most of the cases if data were entered in the whole observation period (2014–2016). The highest symptom severities are either recorded in the same section of the season or changed to the next adjacent category. These profile changes can be explained in most of the cases by the course of the grass pollen season and the changes in the pollination periods of individual grass species. A good example is the comparison of the grass pollen seasons 2014 and 2015. The grass pollen season 2015 in Vienna was warm and dry and most of the grass species flowered together at the same time,^16^ whereas the grass pollen season 2014 was an average season and the pollination periods of the different grass species did not overlap to such an extent.^15^ Only some users did not show a stable pattern and experienced the highest symptoms in every section of the season comparing the 3 years.

### Limitations

The crowd-sourced symptom data used herein are generated from potential, but not medically diagnosed grass pollen allergy sufferers. It remains unknown if users are real patients suffering from pollen allergies since they record symptoms on a voluntary basis. Moreover, a cumulative effect on symptom data due to polysensitization of users could affect the results. Users only enter symptoms if they experience allergic burden. Hence, not all users enter data during the whole time period (2014–2016). Other human factors such as location changes (eg, holiday periods) or days with low pollen concentrations could affect daily user entries as well.

However, the user filtering methods applied before performing the statistical methods decrease these risks to a minimum and the data can be seen as a proxy for allergy symptom data.

## Conclusion

The individual symptom profiles of grass pollen allergy sufferers give insights into the complexity of the grass pollen season and support the explanation of a combined cross-species, multi allergen, system rather than linking symptom profiles with cross reactivity of grass pollen allergens alone. Furthermore, the results of this study show the importance of personal pollen information, as well as phenological monitoring to document the development of different grass species throughout the season. The user profiles of electronic pollen diaries are an interesting additional data source for clinical trials and confirmatory studies of immunotherapy in future. The importance of crowd-sourced symptom data and e-health/m-health services will continue to rise in the future and are a useful complement for scientific studies on human health.

## Abbreviations

ANOVA, Analysis of variance; APIn, Annual pollen integral; EAN, European Aeroallergen Network; PHD, Patient's Hayfever Diary; ZAMG, Zentralanstalt für Meteorologie und Geodynamik.

## Consent for publication

All authors consented for publication in this study. The authors did not publish this research in other articles or journals.

## Authors contribution

The study was designed by MaBa, KB, and UB. Data preparation and analyses were performed by MaBa, MaBe, KB, and LD. Technical and scientific supervision was carried out by MaBe and UB. All authors were involved in data interpretation and drafting, editing and final approval of the manuscript.

## Availability of data and material

All data generated or analyzed during this study is included in this published article. However, users of the Patient's Hayfever Diary were guaranteed anonymity, so no raw data or individual user data is available.

## Ethics approval

Only crowd-sourced and anonymized symptom data were used, so no ethics approval was needed. Moreover, the authors declare that this manuscript complies with the ethics in publishing guidelines.

## Declaration of competing interest

The authors report no competing interests.

## Appendix A Supplementary data

Multimedia component 1 Course of the grass pollen seasons from 2014 to 2016 in Vienna using the EAN standard season definition (1–95% of the cumulative APIN). Multimedia component 1

Multimedia component 2 ANOVA type II tests supporting the significance of the statistical analysis for the calculation with the APIn, the phenology and the SLI. Multimedia component 2
